# Two distinct phenotypes, hemiplegic migraine and episodic Ataxia type 2, caused by a novel common *CACNA1A* variant

**DOI:** 10.1186/s12883-020-01704-5

**Published:** 2020-04-26

**Authors:** Rosaria Nardello, Giorgia Plicato, Giuseppe Donato Mangano, Elena Gennaro, Salvatore Mangano, Filippo Brighina, Vincenzo Raieli, Antonina Fontana

**Affiliations:** 1 Department of Health Promotion, Mother and Child Care, Internal Medicine and Medical Specialities, Specialities “G. D’Alessandro,” University of Palermo, Palermo, Italy; 2 UOC Laboratorio di Genetica Umana, IRCCS Istituto Giannina Gaslini, Genoa, Italy; 3 Department of Experimental Biomedicine and Clinical Neurosciences, University of Palermo, Palermo, Italy; 4 Child Neuropsychiatry Department, Di Cristina - ARNAS Civico Hospital, Palermo, Italy

**Keywords:** *CACNA1A* gene, Familial hemiplegic migraine type 1, Episodic ataxia type2, Cognitive affective syndrome, neuropsychology.

## Abstract

**Background:**

To investigate the genetic and environmental factors responsible for phenotype variability in a family carrying a novel *CACNA1A* missense mutation. Mutations in the *CACNA1A* gene were identified as responsible for at least three autosomal dominant disorders: FHM1 (Familial Hemiplegic Migraine), EA2 (Episodic Ataxia type 2), and SCA6 (Spinocerebellar Ataxia type 6). Overlapping clinical features within individuals of some families sharing the same *CACNA1A* mutation are not infrequent. Conversely, reports with distinct phenotypes within the same family associated with a common *CACNA1A* mutation are very rare.

**Case presentation:**

A clinical, molecular, neuroradiological, neuropsychological, and neurophysiological study was carried out in proband and his carrier mother. The new heterozygous missense variant c.4262G > A (p.Arg1421Gln) in the *CACNA1A* gene was detected in the two affected family members. The proband showed a complex clinical presentation characterized by developmental delay, poor motor coordination, hemiplegic migraine attacks, behavioral dysregulation, and EEG abnormalities. The mother showed typical episodic ataxia attacks during infancy with no other comorbidities and mild cerebellar signs at present neurological evaluation.

**Conclusions:**

The proband and his mother exhibit two distinct clinical phenotypes. It can be hypothesized that other unknown modifying genes and/or environmental factors may cooperate to generate the wide intrafamilial variability.

## Background

Hemiplegic Migraine (HM) is a rare migraine variant characterized by specific aura signs including some degree of hemiparesis and/or one-sided weakness. It may include sensory, visual or language impairment during aura. Two forms of HM have been recognized: familial hemiplegic migraine (FHM MIM #301011), in which at least a relative has migraine aura signs with the same clinical features, and sporadic hemiplegic migraine (SHM) with no recognizable familial history of hemiplegic migraine. FHM is an autosomal dominant inherited disorder caused by mutations in ion transporters encoding several genes: *CACNA1A* mutations cause FHM type 1 (FHM1, MIM:141500), *ATP1A2* mutations cause FHM type 2, *SCNA1* mutations cause FHM type 3 (ICHD-3beta, 2013) [1]. Episodic ataxia type 2 (EA2, MIM: 108500) is a paroxysmal neurological dysfunction of cerebellum lasting minutes to hours that includes symptoms like ataxia, nausea,vomiting, vertigo, diplopia, nystagmus, dysarthria, tinnitus, headache, and hemiplegia. Age of onset ranges between the first and the second decade of life, prevalence rate is unknown. The EA2 attacks can be triggered by some stressors such as alcohol, caffeine, physical and mental stress and postural changes, and relieved by rest and sleep. Neurological examination between attacks may be normal, but some patients may develop ataxia and nystagmus and progressive cerebellar atrophy on MRI. EA2 is an autosomal dominant disorder caused by mutation in the *CACNA1A* gene [2]. Mutations of the *CACNA1A* gene, located on chromosome 19p13.13, coding for the α1 pore –forming subunit of the voltage-gated calcium channel P/Q type (Cav2.1), were identified as responsible for three autosomal dominant disorders with incomplete penetrance: FHM1, EA2, and spinocerebellar ataxia type 6 (SCA6, MIM:183086) [3, 4]. However, overlapping clinical features described within some families with the same *CACNA1A* mutation are not infrequent [5–8]. Conversely, Pradotto reported distinct phenotypes, ranging from EA2 to SCA6, within the same family associated with a common *CACNA1A* mutation [9]. We report a child with a clinical phenotype characterized by some hemiplegic migraine attacks and neurodevelopmental disorder associated with a novel heterozygous c.4262G > A *CACNA1A* (NM_023035.2) variant. The same genetic abnormality was detected only in his mother with clinical history of a distinct infantile EA2 without migraine.

## Case presentation

This study was approved by the ethics committee Palermo 1 of the “Paolo Giaccone” University Hospital of Palermo, Italy. Written informed consent for participate and for publication was obtained from each of the family members tested. The proband is a 7 year old child, the first of two siblings born to apparently healthy unrelated parents at the end of a pregnancy undertaken by an emergency caesarean section, following a gestational period complicated with some bleeding events from the seventh month.

His birth weight was 3900 g and his clinical postnatal course was uneventful. His developmental milestones have been delayed; he vocalized undifferentiated nasal sounds at 12 months, spoke the first word at 2 years and at 13 months he walked unsupported. He is currently under speech and neuropsychomotor therapy.

At the age of 4 years and 8 months, he was referred to our Child Neuropsychiatric Department for unilateral frontal headache attacks associated with pallor, crying, discomfort in the left lower limb, left-sided weakness, mostly in the left lower limb, leading to unsteady posture. The clinical events, started from 4 years of age, occurred almost every day in both wakefulness and sleep, lasted 10 to30 minutes, and spontaneously remitted after resting.

On admission the neurological examination showed a moderate motor and coordination impairment. The cognitive and behavioral evaluation revealed a severe deficit in behavioral regulation (restlessness, poor control of emotional drives with aggressive behavior, poor waiting and following rules abilities) and impaired language with severe phonetic-phonological failings.

**Fig. 1:**
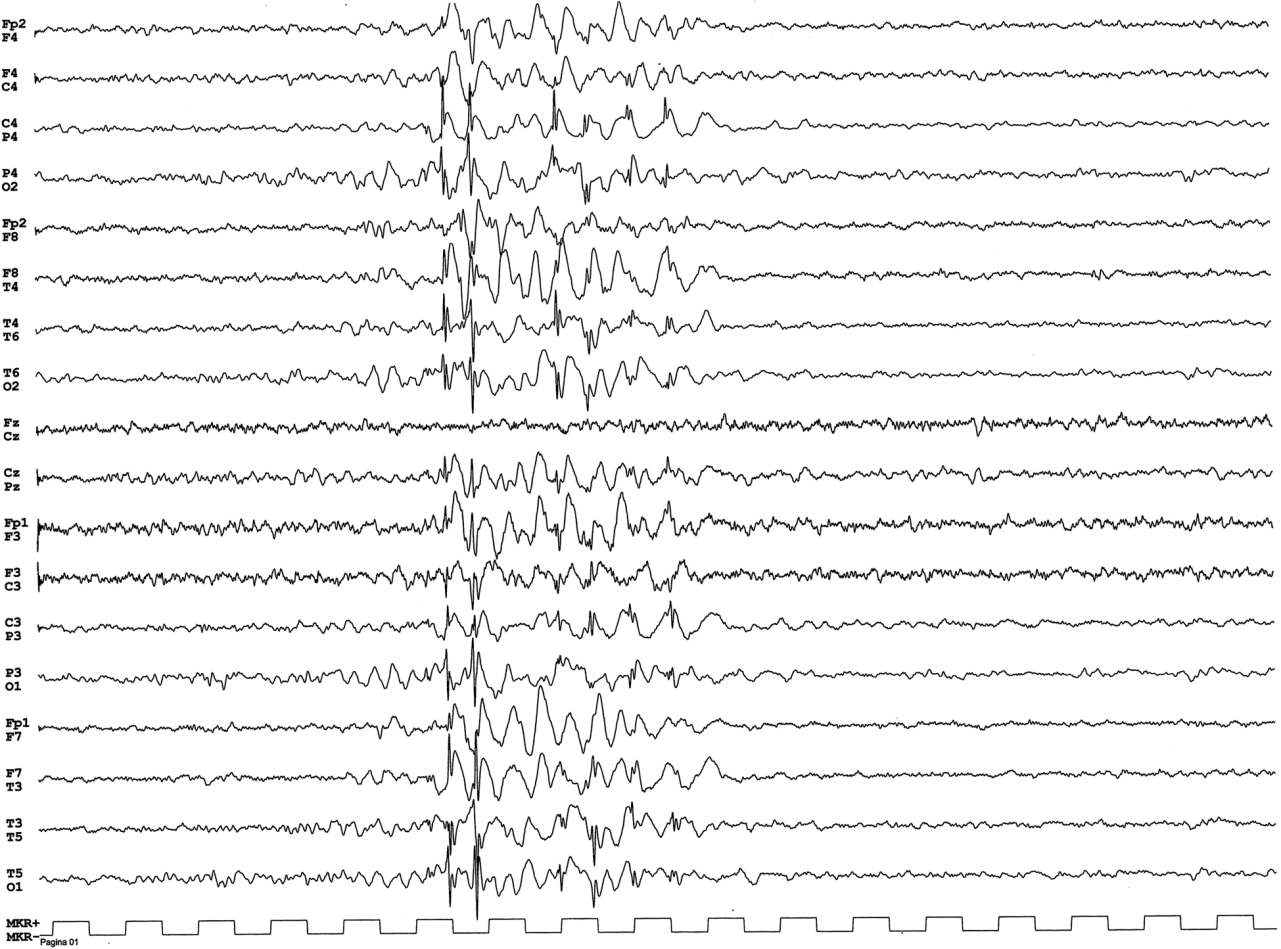
The EEG displayed recurrent generalized 3 Hz regular spike-wave complex discharges lasting 1to 3 s without associated clinical events

Wakefulness and sleep EEG displayed recurrent generalized 3 Hz regular spike-wave complex discharges lasting 1 to 3 s without associated clinical events (Fig. 1).

A treatment of valproic acid with increasing doses up to 30 mg∕ kg/die was started. The electroclinical follow-up displayed a striking disappearance of EEG abnormalities associated with a decrease of hemiplegic migraine attacks and a mild improvement of behavioral symptoms.

The latest clinical evaluations confirmed the disappearance of the generalized 3 Hz spike-wave discharges, showed a decrease of hemiplegic migraine attacks, unchanged motor abnormalities and a worsening of behavioral problems that required an antipsychotic drug in add-on to valproic acid.

The child underwent a comprehensive neuropsychological assessment using a battery of tests standardized for the Italian population (WISC-IV, NEPSY-II, Vineland ABS-II; references were reported in “supplementary material”) to evaluate intellectual, cognitive, and behavioral functions. The formal assessment showed an extremely low overall intellectual ability (IQ = 44) with borderline to average conceptual reasoning and problem-solving skills (PRI 72-88). The performance on measures of expressive vocabulary and lexical knowledge were in the borderline average range. The comprehension of instructions was impaired. The performance on motor dexterity and visuomotor precision was impaired; visuospatial and constructional tasks scored in the average- low average range. The memory tasks for faces and for names fell in the average and below average respectively. The performances on memory for designs and a story memory tasks were impaired in both immediate and delayed free recall. Attention was impaired on auditory tasks and in average range on visual tasks. Executive functions were impaired on inhibition, verbal fluency, and planning tasks. Social cognition (ToM and affect recognition from faces tasks) was impaired (Table 1).

**Table 1:** Neuropsychological test results

WISC-IV				NEPSY II			Vineland ABS-II	
	Score	95% CI	Classification range	Subtests	score	Classification	Scales	Score
Verbal Comprehension Index	56	52–66	Extremely low	**Memory**			Communication	63
Perceptual Reasoning Index	78	72–88	Borderline - average	Memory for faces total	8	average	Daily living skills	77
Working Memory Index	46	43–61	Extremely low	Immediate	9	average	Socialization	79
Processing Speed Index	47	45–65	Extremely low	Delayed	8	average	Motor skills	37
Intelligence Quotient	44	41–53	Extremely low	Memory for names total	6	below average	Adaptive Behavior Composite	56
Cognitive Proficiency Index	33		Extremely low	Delayed	4	impaired		
Fluid Reasoning	63	58–74	Extremely low-borderline	Learning trials	5	below average	Subscales	Age (yr.mo) equivalent
Lexical knowledge	74	68–85	Extremely low-average	Memory for designs				
LTM	69	64–79	Extremely low-borderline	Immediate	1	impaired	Receptive	3.8
STM	46	43–60	Extremely low	Delayed	2	impaired	Expressive	4.8
**Subtests:**	score			Narrative memory			Written	4.3
**Verbal tasks**				Free recall	4	impaired	Personal	4.2
Similarities	2		Impaired	Cued recall	1	impaired	Domestic	5.9
Vocabulary	5		Below average	Recognition	8	average	Community	4.9
Information	4		Impaired	Sentence repetition	1	impaired	Interpersonal relationships	5.6
Comprehension	1		Impaired	**Sensorimotor**			Play and leasure time	4.3
Word Reasoning	6		Below average	Fingertip tapping	1	impaired	Coping skills	4.1
**Perceptual tasks**				Imitating hand position	2	impaired	Gross motor skills	2.9
Block Design	9		Average	Visuomotor precision	4	impaired	Fine motor skills	3.3
Matrix Reasoning	8		Average	**Visuospatial**				
Picture Concepts	3		Impaired	Design coping general	5	below average		
**Working Memory Tasks**				Design coping specific	1	impaired		
Digit Span	1		Impaired	Block construction	6	below average		
Forward	3		Impaired	Geometric puzzle	9	average		
Backward	3		Impaired	**Language**				
Arithmetic	2		Impaired	Phonological processing	4	impaired		
Letter-Number sequencing	1		Impaired	Speeded naming	8	average		
**Processing Speed Tasks**				Comprehension of instructions	1	impaired		
Coding	1		Impaired	Semantic fluency	1	impaired		
Symbol Search	1		Impaired	Phonological fluency	2	impaired		
				**Attention/Executive**				
				Auditory attention	<2%ile	impaired		
				Visual attention	9	average		
				Design fluency	6	below average		
				Inhibition	1	impaired		
				**Social perception**				
Score: Standard score (mean100, standard deviation 15)				Theory of mind	4	impaired		
Score: Scaled score (mean 10, standard deviation 3)				Affect recognition	4	impaired		

The child showed difficulties in emotion regulation including easy frustration, temper tantrums, oppositional behaviors, verbally and physically aggressive behavior.

MRI investigation, performed at 5 years of age, did not show structural abnormalities. The more detailed family clinical history highlighted some paroxysmal events in mother childhood with onset at 8 years of age characterized by sudden dramatic gait unsteadiness, vertigo, nausea and sometimes vomiting. These attacks occurred several times a week and lasted for hours, they were triggered by exertion and postural changes and relieved by rest and sleep. At the age of 15 interictal neurological and vestibular evaluation, and a cranial CT scan were not informative regarding the disorder cause. Although no treatment was recommended, the frequency of attacks graduallydecreased until their remission. In addition, the mother firmly denied a family history of FHM1 or EA2 attacks and SCA6. A recent neurological evaluation revealed nystagmus by lateral gaze, moderate fine motor impairment, dysdiadochokinesia. The intellectual assessment showed an extremely low overall intellectual ability (WAIS IV IQ = 49). General adaptive functioning was in the average compared to age matched peers (Vineland II: Adaptive Behavior Composite = 90) and she engages in a work activity and takes care of her family. The clinical and EEgraphic assessment, extended to the sister and the father of proband, showed neither migraine and / or cerebellar symptoms, nor cognitive and behavioral impairment, nor the 3 Hz SW pattern found in the patient.

**Fig. 2:**
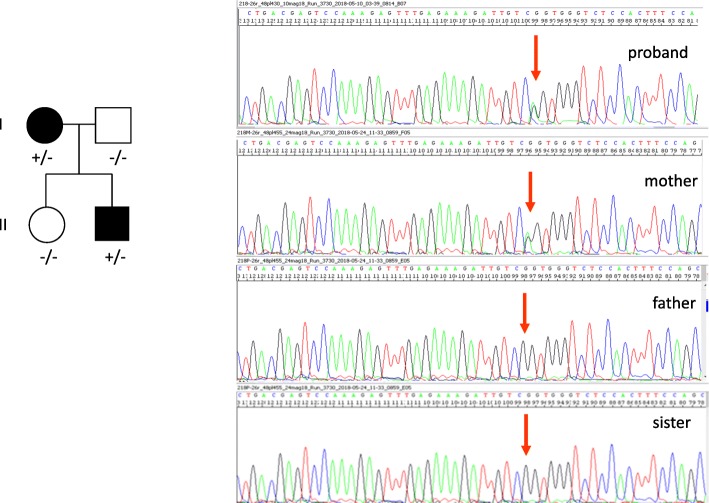
The novel mutation at c.4262G > A, p.(Arg1421Gln) in the CACNA1A gene was found in the proband and his mother

A next generation sequencing panel, exploring 45 genes associated with epileptic encephalopathy (for more details see the “supplementary material”), performed in the proband and his family members, showed a new heterozygous missense variant, c.4262G > A (p.Arg1421Gln) in the *CACNA1A* gene (NM_023035.2), [Chr19(GRCh37):g.13372264C > T], inherited from his mother, never described in literature and not reported in gnomAD and ExAC database (Fig. 2).

This variant was not been found in his healthy father and his healthy sister. This substitution causes the replacement of a positively charged arginine with a neutral not polar glutamine and involves a highly conserved residue located in the extracellular pore loop connecting S5 and S6, likely altering the calcium channel structure at this functionally critical region. A computer-based analysis, able to predict the effect of amino acid change on protein structure and function by using three different algorithms, PolyPhen2(http://genetics.bwh.harvard.edu/pph2/), Mutation taster http://www.mutationtaster.org/, and HSF (https://umd.be › HSF) respectively, indicated a possible deleterious effect on protein function (possibly damaging -score 0,6- for PolyPhen2, disease causing -score 43- for Mutation taster, and potential alteration of splicing for HSF). In addition, the variant, located very close to the exon-intron boundary, could lead to an abnormal splicing resulting in aberrant transcripts (Alamut visual ese predictions: Splicing predictions at nearest natural junction predicted change at donor site 1 bps downstream: − 64.2% MaxEnt: -81.2%, NNSPLICE: -98.9%, HSF: − 12.6%).

## Discussion and conclusions

The neuronal Ca_V_ channels control several cellular functions and are involved in some neurological and psychiatric disorders [10].

The Cav2.1 channels are localized at presynaptic nerve terminals where the depolarization induces voltage-dependent calcium influx mediating the neurotransmitter release. They are expressed in all brain regions including the thalamocortical system, where P/Q-type VGCCs support the production of characteristic γ-band oscillations considered to be a functional prerequisite to cognitive states, but they are particularly expressed in Purkinje and granule cells [11, 12]. The identification of the pathogenic role of *CACNA1A* mutations in some disorders such as EA2, FHM1, and SCA6 suggested that these may be considered allelic disorders [3, 4]. Earlier it was hypothesized that the mutations inducing loss-of-function were associated with EA2, missense mutations resulting in gain-of-function caused FHM1, and abnormal CAG expansions were related with SCA6 [3, 4]. But overlapping features, including typical and uncommon symptoms of mentioned disorders, have been reported among the members of the same family [5–8]. Recently, a common *CACNA1A* mutation with distinct phenotypes, ranging from epileptic encephalopathy, EA2 to SCA6, was also found within the same family, suggesting that several factors could play a role in the phenotypic variability [9, 13].

Our affected family members share the same heterozygous c.4262G > A *CACNA1A* nucleotide exchange, located very close to the exon-intron boundary, which leads to the p.Arg1421Gln missense mutation. This variant is not included in the gnomAD browser and never identified in patients with FHM1, EA2, SCA6 with whom, therefore, a comparison is impossible. Nevertheless, it has been reported that most FHM1 and EA2 missense mutations produce substitutions of conserved amino acids (frequently arginine) clustering in important functional regions of the Ca_v_2.1 channel including the pore lining (FHM1), the voltage sensors (FHM1), and the S5–S6 linkers (EA2) [12, 14, 15]. In addition, the p.Arg1421Gln variant segregating in all our affected family members and sparing the asymptomatic father and sister enhances its pathogenic relevance. The two patients showed two distinct phenotypes, the mother exhibited the typical EA2 without other comorbidities, and her son developed a more complex clinical feature characterized by hemiplegic migraine attacks, developmental delay, poor developmental movement coordination, and behavioral dysregulation. Since no relative has migraine aura symptoms, our child’s clinical feature could be classified as SHM with early onset. This co-occurrence in *CACNA1A* mutant patients has been associated with more severe outcome [16]. Many studies remarked in FHM1, EA2,and SCA6 patients the co-occurrence of behavioral and neuropsychological disorders but the investigations carried out with formal neuropsychological testing are scarce [17–19]. Recently, a large case series of 23/44 patients (11/23 FHM1,10/23 EA2, 2/23 SCA6), investigated by a formal neuropsychological testing, predominantly showed figural memory, visuoconstructive abilities, and verbal fluency impairment. Furthermore, the study revealed that neuropsychological deficits were consistent with history of development delay of patients [19]. A case study carried out using a formal neuropsychological assessment reported an impairment of semantic fluency and of processing speed, and a mild executive dysfunction [18]. A careful evaluation of our patient using a comprehensive neuropsychological battery revealed a partial agreement with the results of Indelicato and Trahan. Indeed, we pointed out intellectual disability and motor skill disorders but the lack of visual constructive disturbances. This discrepancy could depend more on the higher sensitivity of the WISC IV and NEPSY II than on the diagnostic tools used in above studies. We assume that the behavioral symptoms of our patient, irrespective of the lack of cerebellum atrophy, may depend on cerebellar dysfunction, being the Cav2.1 channel greatly expressed in the cerebellum. These behavioral symptoms could be comparable to the clinical features characterizing the well-known cerebellar cognitive affective syndrome [20–22]. Development delay of the child suggests also an impairment of cognitive networks due, besides the cerebellar dysfunction, also to an abnormal hippocampal neurotransmission as documented in knock-in mice model expressing the FHM1 mutation [23].

In addition, the child showed an electroencephalographic pattern characterized by frequent but short generalized 3 Hz regular spike-wave discharges without evidence of ictal clinical events. Nevertheless, their disappearance after valproic acid treatment associated with a mild improvement of attention and behavioral symptoms suggested that the child for an indefinite time may have suffered from “micro absence” and/or a mild cognitive impairment. This finding agrees with the electroclinical pattern often highlighted in EA2 patients carrying a truncating mutation of *CACNA1A*, suggesting a role of calcium channels in absence epilepsy although the electroclinical pattern in our patient is associated with the FHM1 phenotype [24, 25].

The impressive behavioral feature found in our patient and in many subjects previously reported in literature suggests the possibility to further expand the phenotype of *CACNA1A* mutations beyond ataxic and hemiplegic symptoms including cognitive and behavioral symptoms.

The careful analysis of our phenotypes does not provide useful data to elucidate the molecular mechanisms underlying the different clinical expressions of the disorder in mother and son.

Although the *CACNA1A* gene can generate different Cav channels by alternative pre-mRNA splicing, some of which have been associated with SCA6 and FHM1 phenotypes, to our knowledge no alternative splicing has been identified to be the cause of intrafamilial phenotypic variability [10, 26]. Furthermore, it has been suggested that mutations involving the splice site regions are often associated with higher variability of the phenotype and with the overlap of the common symptoms of the allelic disorders (FHM1 and SCA6) [17]; in addition, the different expression of Ca_v_2.1 in the several neural networks could account for the variability of symptoms among the family members carrying the same mutation. Finally, as the emotional stress, minor head trauma, physical exercise, alcohol or caffeine are the most common triggers of FHM1 and EA2 attacks, other unknown individual modifier genes and / or environmental factors could play a regulator role in the phenotypic variability.

## Data Availability

All data generated or analyzed during current study are available from the corresponding author on reasonable request.

## Abbreviations

HM: Hemiplegic migraine
SHM: Sporadic hemiplegic migraine
FHM: Familial hemiplegic migraine
CACNA1A: Calcium channel, voltage-dependent, p/q type, alpha-1a subunit
ATP1A2: ATPase Na+/K+ transporting subunit alpha 2
SCNA1: Sodium channel, neuronal type i, alpha subunit
EA2: Episodic ataxia type 2
SCA6: Spinocerebellar ataxia type 6
EEG: Electroencephalogram
WISC-IV: Wechsler intelligence scale IQ score
NEPSY-II: Developmental NEuroPSYchological Assessment
ToM: Theory of Mind
ExAC: Exome Aggregation Consortium
gnomAD: Genome Aggregation Database
PolyPhen2: Polymorphism phenotyping v2
HSF: Human splicing finder
CT: Computed tomography
WAIS: Wechsler Adult intelligence scale IQ score
Cav2.1: P/Q voltage-dependent calcium channel
Vineland ABS-II: Vineland adaptive behavior scales-ii
VGCCs: Voltage-gated calcium channels
CAG: Repeated codon CAG
